# Single and co-infections by *Anaplasma platys*, *Babesia vogeli*, *Ehrlichia canis*, and *Hepatozoon canis* in dogs from Rio Grande do Norte, Brazil: frequency and molecular characterization

**DOI:** 10.1590/S1984-29612025067

**Published:** 2025-12-19

**Authors:** Bruno Vinicios Silva de Araújo, Isa Lorena Pinto Dantas Bezerra, Gabriela Linhares Leite, Amanda Haisi, João Pessoa Araújo, Francisco de Assis Leite Souza, Leucio Camara Alves, João Marcelo Azevedo de Paula Antunes, Juliana Fortes Vilarinho Braga

**Affiliations:** 1 Universidade Federal Rural de Pernambuco – UFRPE, Programa de Pós-graduação em Biociência Animal, Recife, PE, Brasil; 2 Universidade Federal Rural do Semi-Árido – UFERSA, Centro de Ciências Agrárias, Departamento de Ciência Animal, Mossoró, RN, Brasil; 3 Universidade Estadual Paulista Júlio de Mesquita Filho – UNESP, Instituto de Biociências e Instituto de Biotecnologia (IBTEC), Botucatu, SP, Brasil; 4 Universidade Federal Rural de Pernambuco – UFRPE, Departamento de Morfologia e Fisiologia Animal, Recife, PE, Brasil; 5 Universidade Federal Rural de Pernambuco – UFRPE, Departamento de Medicina Veterinária, Recife, PE, Brasil; 6 Universidade Federal Rural do Semi-Árido – UFERSA, Centro de Ciências Agrárias, Hospital Veterinário Universitário, Mossoró, RN, Brasil; 7 Universidade Federal do Piauí – UFPI, Colegiado do Curso de Graduação em Medicina Veterinária, Bom Jesus, PI, Brasil

**Keywords:** hemoparasitosis, tick-borne diseases, *Rhipicephalus sanguineus* sensu lato (s.l.), PCR, phylogeny, molecular epidemiology, hemoparasitoses, doenças transmitidas por carrapatos, *Rhipicephalus sanguineus* sensu lato (s.l.), PCR, filogenia, epidemiologia molecular

## Abstract

This study investigated the presence of *Anaplasma platys*, *Babesia vogeli*, *Ehrlichia canis*, and *Hepatozoon canis* in dogs in Mossoró, Rio Grande do Norte, Brazil, from October 2022 to August 2023. Blood samples were collected from 181 dogs with suspected hemoparasitoses based on clinical and/or laboratory findings (such as hematological alterations) for direct hemoparasite examination on slides, polymerase chain reaction (PCR), and phylogenetic analysis. PCR revealed that 72.3% of dogs were infected with at least one agent. Among these, 44.7% were monoinfected and 27.6% coinfected, with 21.5% infected by two agents and 6.1% by three or more. *E. canis* was most frequent (41.9%), followed by *H. canis* (35.4%), *A. platys* (21.5%), and *B. vogeli* (7.7%). *Anaplasma* and *Ehrlichia*-like morulae, *Hepatozoon* sp. gametocytes, and *Babesia* sp. merozoites were found in 20.0%, 11.1%, 15.0%, and 1.7% of animals, respectively. Phylogenetic analysis of 16S and 18S rRNA gene sequences revealed high similarity to reference strains from Brazil and other countries. The infection patterns underscore the importance of diagnostic and preventive measures to safeguard animal health in areas endemic for *Rhipicephalus sanguineus* sensu lato (s.l.), given the frequency of coinfections that may exacerbate clinical signs and hinder treatment.

## Resumo

Este estudo investigou a presença de *Anaplasma platys*, *Babesia vogeli*, *Ehrlichia canis* e *Hepatozoon canis* em cães de Mossoró, Rio Grande do Norte, Brasil, entre outubro de 2022 e agosto de 2023. Amostras sanguíneas de 181 cães sob suspeita clínica e/ou laboratorial (como alterações hematológicas) de hemoparasitoses, foram analisadas por pesquisa direta em lâmina, reação em cadeia da polimerase (PCR) e análise filogenética. A PCR revelou infecção por ao menos um agente em 72,3% dos cães. Desses, 44,7% apresentaram monoinfecção e 27,6% coinfecção, sendo 21,5% por dois agentes e 6,1% por três ou mais. *Ehrlichia canis* foi o agente mais frequente (41,9%), seguido por *H. canis* (35,4%), *A. platys* (21,5%) e *B. vogeli* (7,7%). Mórulas similares às de *Anaplasma* sp. e *Ehrlichia* sp., gametócitos de *Hepatozoon* sp. e merozoítos de *Babesia* sp. foram observados em 20,0%, 11,1%, 15,0% e 1,7% dos animais, respectivamente. A análise filogenética dos genes 16S rRNA e 18S rRNA revelou alta similaridade às amostras do Brasil e do mundo. Os padrões de infecção observados destacam a importância de medidas diagnósticas e preventivas em áreas endêmicas para *Rhipicephalus sanguineus* sensu lato (s.l.), dada a frequência de coinfecções que podem agravar os sinais clínicos e dificultar o tratamento.

## Introduction

Emerging and re-emerging vector-borne diseases pose significant challenges to veterinary and human medicine (Fonsêca et al., 2022), underscoring their importance to global health (Oliveira et al., 2020). Despite there being over 900 identified tick species globally, roughly 10% of them rank just behind mosquitoes as the primary vectors responsible for transmitting diseases to humans and animals (Tabor, 2024). In Brazil, the occurrence of tick species is highly variable, with their presence in each locality being determined by the epidemiological characteristics of the region (Labruna & Pereira, 2001; Labruna et al., 2005).

Among the infectious agents transmitted by ixodid ticks frequently reported in the Brazilian territory affecting dogs, we highlight the *Ehrlichia canis* and *Anaplasma platys* bacteria and *Babesia vogeli* and *Hepatozoon canis* protozoa (Malheiros et al., 2016; Guimarães et al., 2021).

These hemoparasites cause various nonspecific clinical signs in infected hosts (Gonçalves et al., 2014; Bouzouraa et al., 2017), which can become more severe depending on the dog's immune status and the presence of coinfections (Movilla et al., 2017). Considering the importance and clinical similarity of these diseases, an early and accurate diagnosis is crucial for implementing appropriate treatment measures and achieving a favorable prognosis (Rucksaken et al., 2019; Guimarães et al., 2021).

Microscopic diagnosis of hemoparasitosis is commonly employed in clinical practice; however, its sensitivity is low and varies depending on the disease stage and experience of laboratory professionals (Kaewkong et al., 2014; Buddhachat et al., 2020; Kaur et al., 2020). Given these limitations, molecular methods, such as polymerase chain reaction (PCR), stand out owing to their high sensitivity and specificity, enabling diagnosis across different stages of infection and accurate identification of various pathogens (Massetti et al., 2020; Silva et al., 2021).

The climatic aspects of the Brazilian semi-arid region, characterized by irregular temporal and spatial rainfall patterns and high annual average temperatures (Lemos et al., 2021), favour the spread of ixodid vectors, particularly *Rhipicephalus sanguineus* sensu lato (s.l.), which are less dependent on humid environments for survival (Tian et al., 2022) and are epidemiologically important in the transmission of hemoparasites in Brazil (Gray et al., 2013). Notably, Šlapeta et al. (2021) have recently established the foundation for using *Rhipicephalus linnaei* to refer to the 'tropical lineage' of *R. sanguineus* s.l..

Numerous cases of tick-borne agents infecting dogs have been reported in Brazil (Melo et al., 2016; Mongruel et al., 2018; Guimarães et al., 2021; Vieira et al., 2021; Bittencourt et al., 2022; Fonsêca et al., 2022). Despite favorable climatic conditions for the development of *R. sanguineus* s.l., studies on the occurrence and characterization of pathogens transmitted by this vector to dogs in the northeastern semi-arid region are scarce. Therefore, this study aimed to determine the frequency of *A. platys*, *E. canis*, *B. vogeli*, and *H. canis* in dogs in an area endemic for *R. sanguineus* s.l. in Brazil and to perform molecular characterization of these pathogens.

## Materials and Methods

### Study area and sample definition

This study was conducted in the municipality of Mossoró (5°11'15''S, 37°20'39''W), located in the semi-arid climate of the state of Rio Grande do Norte, Brazil. According to the Köppen climate classification, this municipality is characterized by a tropical rainy climate, with a dry winter and a rainy season in the summer. It has an average annual temperature of 27.4°C, irregular annual rainfall, and an average relative humidity of 68.9% (Nogueira et al., 2024).

In this study, the inclusion criteria were domestic dogs of any sex or breed, aged two months or older, presenting clinical signs and/or laboratory evidence suggestive of infection by *A. platys*, *E. canis*, *B. vogeli*, and/or *Hepatozoon canis* observed by the attending veterinarian (lethargy, anorexia, fever, hyporexia, weight loss, dehydration, pale mucous membranes, petechiae, epistaxis, lymphadenomegaly, thrombocytopenia, and/or anemia), associated with the presence or history of exposure to ticks. Dogs younger than two months were explicitly excluded from the study. All samples were obtained from dogs attended at public and popular hospitals in the city, of which 55 dogs came from the public hospital and 126 from popular hospitals. In this context, 'public' refers to a government-funded veterinary hospital that provides free care, whereas 'popular' refers to privately owned, low-cost veterinary clinics that offer affordable services to the general population. The study employed a convenience sampling strategy, selecting dogs based on clinical and/or laboratory suspicion of tick-borne infections. All procedures were approved by the Ethics Committee on Animal Use under the protocol number described in the specific section at the end of this manuscript.

### Dog blood collection

Blood samples were collected by cephalic or jugular venipuncture with proper antiseptic precautions and were placed in tubes containing ethylenediaminetetraacetic acid (EDTA) at 4°C for DNA extraction. Blood smear slides were prepared, stained using the rapid panoptic (RenyLab®, Brazil) method for hemoparasite screening, and examined at 1000× magnification using a Nikon Eclipse E-200 microscope (Nikon®, Japão), as described by Kerr (2003). The entire length of the blood smear was examined during the analysis.

### DNA extraction and PCR

DNA was extracted from the blood samples using a PureLink® Genomic DNA Mini Kit (Thermo Fisher Scientific®, Waltham, MA, USA), following the manufacturer's recommendations. Following extraction, the genomic material was quantified using a spectrophotometer (NanoDrop™ Lite; Thermo Fisher Scientific®), and the samples were stored at -20 °C until PCR was conducted.

The dog β-actin gene was used as the endogenous control for DNA extraction. Specific target genes, oligonucleotide primers, and validated amplification programs from previous studies were used to detect *A. platys, E. canis, B. vogeli* and *H. canis* (Table 1).

**Table 1:** Oligonucleotide sequences, amplification programs, and PCR product sizes for canine beta-actin, *Anaplasma platys*, *Ehrlichia canis*, *Babesia vogeli*, and *Hepatozoon canis* used in this study.

**Pathogen (gene target)**	**Primer**	**Oligonucleotide Sequence (5’-3’)**	**Amplification program**	**Product (bp)**	**Reference**
Canine β-actin	Actb-F	GGCATCCTGACCCTGAAGTA	Initial Denaturation: 95°C, 10’ 35 cycles of: Denaturation: 95°, 30’’ Annealing: 60°C, 30’’ Extension: 72°C, 30’’ Final Extension: 72°C, 5’	98pb	Turchetti (2014)
	Actb-R	CGCAGCTCGTTGTAGAAGGT			
*Anaplasma platys* (16S rRNA)	EPLAT5-F	TTTGTCGTAGCTTGCTATGAT	Initial Denaturation: 95°C, 5’ 40 cycles of: Denaturation: 94°, 30’’ Annealing: 58°C, 30’’ Extension: 72°C, 45’’ Final Extension: 72°C, 5’	386pb	Matei et al. (2016), with modifications
	EPLAT3-R	CTTCTGTGGGTACCGTC			
*Ehrlichia canis* (16S rRNA)	EHO-F	AGAACGAACGCTGGCGGCAAGCC	Initial Denaturation: 94°C, 10’ 40 cycles of: Denaturation: 94°, 60’’ Annealing: 60°C, 60’’ Extension: 72°C, 60’’ Final Extension: 72°C, 4’	478pb	Bulla et al. (2004)
	EHO-R	CGTATTACCGCGGCTGCTGGC			
	ECA-F	CAATTATTTATAGCCTCTGGCTATAGGAA	Initial Denaturation: 94°C, 10’ 40 cycles of: Denaturation: 94°, 60’’ Annealing: 60°C, 60’’ Extension: 72°C, 60’’ Final Extension: 72°C, 4’	389pb	
	ECA-R	TATAGGTACCGTCATTATCTTCCCTAT			
*Babesia vogeli* (18S rRNA)	BAB1	GTGAACCTTATCACTTAAAGG	Initial Denaturation: 94°C, 2’ 35 cycles of: Denaturation: 94°, 30’’ Annealing: 56°C, 30’’ Extension: 72°C, 60’’ Final Extension: 72°C, 5’	602pb	Duarte et al. (2008)
	BAB4				
		CAACTCCTCCACGCAATCG			
*Hepatozoon canis* (18S rRNA)	HC-18S-F	CACCAGGTCCAGACATAGAAAG	Initial Denaturation: 95°C, 3’ 35 cycles of: Denaturation: 95°, 30’’ Annealing: 62°C, 30’’ Extension: 72°C, 45’’ Final Extension: 72°C, 1’	306pb	Kaur et al. (2020)
	HC-18S-R	AAGCTTACCAGCCAAGGTTAT			

The PCRs were conducted in a final volume of 25 μL, comprising 12.5 μL of master mix (Platinum PCR SuperMix; Cellco®, Brazil), 1 μL of each primer (10 µM), 9.5 μL of diethyl pyrocarbonate (DEPC)-treated water, and 1 µL of DNA. For the amplification of *E. canis* DNA, nested PCR was performed, using 1 μL of the product from the first PCR step as a template for the second reaction. Amplification of *A. platys*, *B. vogeli*, and *H. canis* DNA was performed via conventional PCR. For all reactions, known positive DNA samples from the agents under investigation were used as positive controls (*E. canis* – GenBank accession number PV354097, *A. platys* – PV354061, *B. vogeli* – PV354366, and *H. canis* – PV354381) and DEPC-treated water was used as a negative control.

All PCR products were subjected to 1.5% agarose gel electrophoresis in 1× Tris-borate-EDTA buffer for 50 min at 120 V, using GelRed® as the DNA stain. A 100 bp DNA ladder (Ludwig®, Brazil) was used as a molecular weight marker, following the manufacturer's recommendations, to determine the size of amplified products. Following electrophoresis, the gel was visualized on a UV transilluminator (ProteinSimple®, CA, USA) and imaged using an AlphaImager Mini System software. Samples were considered positive for *A. platys*, *E. canis*, *B. vogeli*, or *H. canis* if they showed amplified products of approximately 386, 389, 602, and 306 bp, respectively.

### Sequencing and phylogenetic analysis

The PCR products were purified using magnetic beads (SpeedBead Magnetic Carboxylate Modified Particles, Azide 0.05%; GE Healthcare® UK Limited, Little Chalfont, Buckinghamshire, England), and their concentration and purity were assessed via spectrophotometry (NanoDrop™ Spectrophotometer; Thermo Fisher Scientific®). Sequencing was conducted on the genetic analyzer 3500 (Applied Biosystems®, Foster City, CA, USA) using the Sanger method, employing specific bidirectional primers for each species (Table 1).

The sense and antisense sequences were trimmed and assembled using Geneious Prime (version 2020.2.1) software. Subsequently, they were compared with other sequences deposited in the GenBank® nucleotide database using the BLAST® nucleotide tool to determine the percentage of identity. Genetic sequences were aligned with known sequences obtained from GenBank using the MAFFT tool (v7.520) (Katoh & Standley, 2013). For *A. platys* and *E. canis*, 32 sequences from the Anaplasmataceae family were included; for *B. vogeli*, 13 sequences from the genus *Babesia*; and for *H. canis*, 14 sequences from the genus *Hepatozoon*. A maximum likelihood phylogenetic tree was constructed using IQ-TREE version 2.2.0.3 (Minh et al., 2020), applying the best-fit models determined by ModelFinder (Kalyaanamoorthy et al., 2017): K2P+I for *A. platys* and *E. canis*, HKY+F for *B. vogeli*, and K2P for *H. canis*. Bootstrap analysis was performed with 1,000 replicates for all phylogenetic reconstructions. Visualization and editing of the reconstruction were performed using FigTree software.

### Statistical analysis

The data are presented as simple frequencies and percentages obtained using the statistical software SAS System for Windows 9.0 (SAS Institute, Cary, NC, USA). Chi-square tests or Fisher’s exact tests were used to assess the association between pathogen positivity and different infection types, specifically multiply infection, co-infection, and mono-infection, with statistical significance set at *p* < 0.05. Fisher's exact test was used when the expected frequency was less than 5.

## Results

All 181 samples subjected to PCR for detection of the dog beta-actin gene yielded an amplified product of approximately 98 bp, confirming their positivity for the gene and validating the viability of the genomic material used.

*Ehrlichia canis* was the most frequently detected agent via PCR in this study (41.9%, 76/181), followed by *H. canis* (35.4%, 64/181), *A. platys* (21.5%, 39/181), and *B. vogeli* (7.7%, 14/181) (Table 2).

**Table 2:** Frequency of PCR positivity for *Anaplasma platys*, *Ehrlichia canis*, *Babesia vogeli*, and *Hepatozoon canis* associated with infection types^¥^ in naturally infected, hospital-treated dogs in a semi-arid region of Brazil in 2023.

**Pathogen PCR positivity**	**Multi-infection (N=11)**		**Co-infection (N=39)**		**Mono-infection (N=81)**		**p-value**
	**Freq.**	**%**	**Freq.**	**%**	**Freq.**	**%**	
PCR(+)^1^*Anaplasma platys*	10	90.9	18	46.2	11	13.6	<0.001**
PCR(+) *Ehrlichia canis*	10	90.9	25	64.1	41	50.6	0.022**
PCR(+) *Babesia vogeli*	06	54.5	06	15.4	02	2.5	<0.001**
PCR(+) *Hepatozoon canis*	08	72.7	29	74.4	27	33.3	<0.001*

* Statistical significance (*p*<0.05, chi-square test);

** Statistical significance (*p*<0.05, Fisher's Exact Test);

¥ Category “Negatives” was excluded from the analysis;

1 PCR(+): PCR positive.

Monoinfection by the pathogens studied was detected in 44.7% (81/181) of the samples. Among these, *E. canis* DNA was detected in 22.6% (41/181) of the animals and had the highest frequency, followed by *H. canis* in 14.9% (27/181), *A. platys* in 6.1% (11/181), and *B. vogeli* in 1.1% (2/181).

Of the analyzed dogs, 21.5% (39/181) were coinfected for two agents, with coinfection by *E. canis* and *H. canis* being detected in 8.8% (16/181) of the animals and was thus the most common, followed by coinfections with *A. platys* and *H. canis* in 6.6% (12/181), *A. platys* and *E. canis* in 2.8% (5/181), *E. canis* and *B. vogeli* in 2.2% (4/181), *A. platys* and *B. vogeli* in 0.5% (1/181), and *B. vogeli* and *H. canis* in 0.5% (1/181) of the dogs. These results indicate that 84.6% (33/39) of the dogs coinfected with two agents were concurrently infected with both bacterial and protozoa.

Among the dogs coinfected with three or more agents (multiply infected), the most frequent was *E. canis* with *A. platys* and *H. canis*, which was detected in 2.8% (5/181) of the cases, followed by *B. vogeli* with *A. platys* and *E. canis* in 1.7% (3/181), *E. canis* with *B. vogeli* and *H. canis* in 0.5% (1/181), and *A. platys* with *B. vogeli* and *H. canis* in 0.5% (1/181). One animal (0.5%) was infected with all the four pathogens.

Regarding the direct examination of agents in blood smears (Table 3), *Anaplasma-*like morulae were observed in 20% (36/180) and *Hepatozoon* sp. gametocytes in 15% (27/180) of the analyzed dogs. *Ehrlichia-*like morulae were observed in 11.11% (20/180) and *Babesia* sp. merozoites in 1.67% (3/180) of the animals. The frequencies of animals that tested positive in the blood smear examination but were not molecularly confirmed via DNA detection of *A. platys*, *H. canis*, *E. canis*, or *B. vogeli* were 20.4% (10/49), 6.1% (3/49), 4.1% (2/49), and 2% (1/49), respectively.

**Table 3:** Frequency of positivity for *Anaplasma* sp., *Ehrlichia* sp., *Babesia* sp., and *Hepatozoon* sp. in direct blood smear examination associated with infection types^1^, in naturally infected, hospital-treated dogs in a semi-arid region of Brazil in 2023.

**Variables**	**Multi-infection (N=11)**		**Co-infection (N=39)**		**Mono-infection (N=81)**		**Negative (N=49)**		**p- value**
	**Freq.**	**%**	**Freq.**	**%**	**Freq.**	**%**	**Freq.**	**%**	
BS(+)^2^ for *Anaplasma* sp.	04	36.4	11	28.2	11	13.6	10	20.4	0.111
BS(+) for *Ehrlichia* sp.	03	27.3	05	12.8	10	12.3	02	4.1	0.105
BS(+) for *Babesia* sp.	0	0.0	0	0.0	02	2.5	01	2.0	1.0
BS(+) for *Hepatozoon* sp.	02	18.2	10	25.6	12	15.0	03	6.1	0.071

Statistical significance (*p*<0.05, Fisher's Exact Test).

1 Type of infection determined via PCR.

2 BS(+): positive blood smear.

Analysis of the association between PCR positivity and infection type revealed that all studied pathogens (*Anaplasma platys*, *Ehrlichia canis*, *Babesia vogeli*, and *Hepatozoon canis*) were significantly associated with mono-, co-, and multiple infections (coinfected with three or more agents) (Table 2) in comparison to the positivity of agents in blood smears, which showed no association with different infection profiles (Table 3).

Ten samples of each studied agent were randomly selected for genetic sequencing, all of which originated from monoinfection cases. The alignment results revealed that all sequences obtained in this study were identical among the amplicons of each pathogen. Therefore, only one sequence per agent was deposited in GenBank. A 100% identity was observed between the consensus sequence and sequences obtained from GenBank using a BLASTn search (GenBank: *A. platys* CP046391.1, *E. canis* KC479023.1, *B. vogeli* MK881128.1 and *H. canis* AY461378). Partial sequences of the 16S (*E. canis* and *A. platys*) and 18S (*B. vogeli* and *H. canis*) rRNA gene obtained in this study were deposited in GenBank under the accession numbers PP211477, PP211197, PP211446, and PP211449, respectively.

Chromatogram analysis of the *Hepatozoon canis* sequences revealed that 9 of the 10 selected *H. canis* samples exhibited double peaks at one site at position 1873 (adenine/guanine). These sequences were not deposited in the GenBank database.

**Figure 1:**
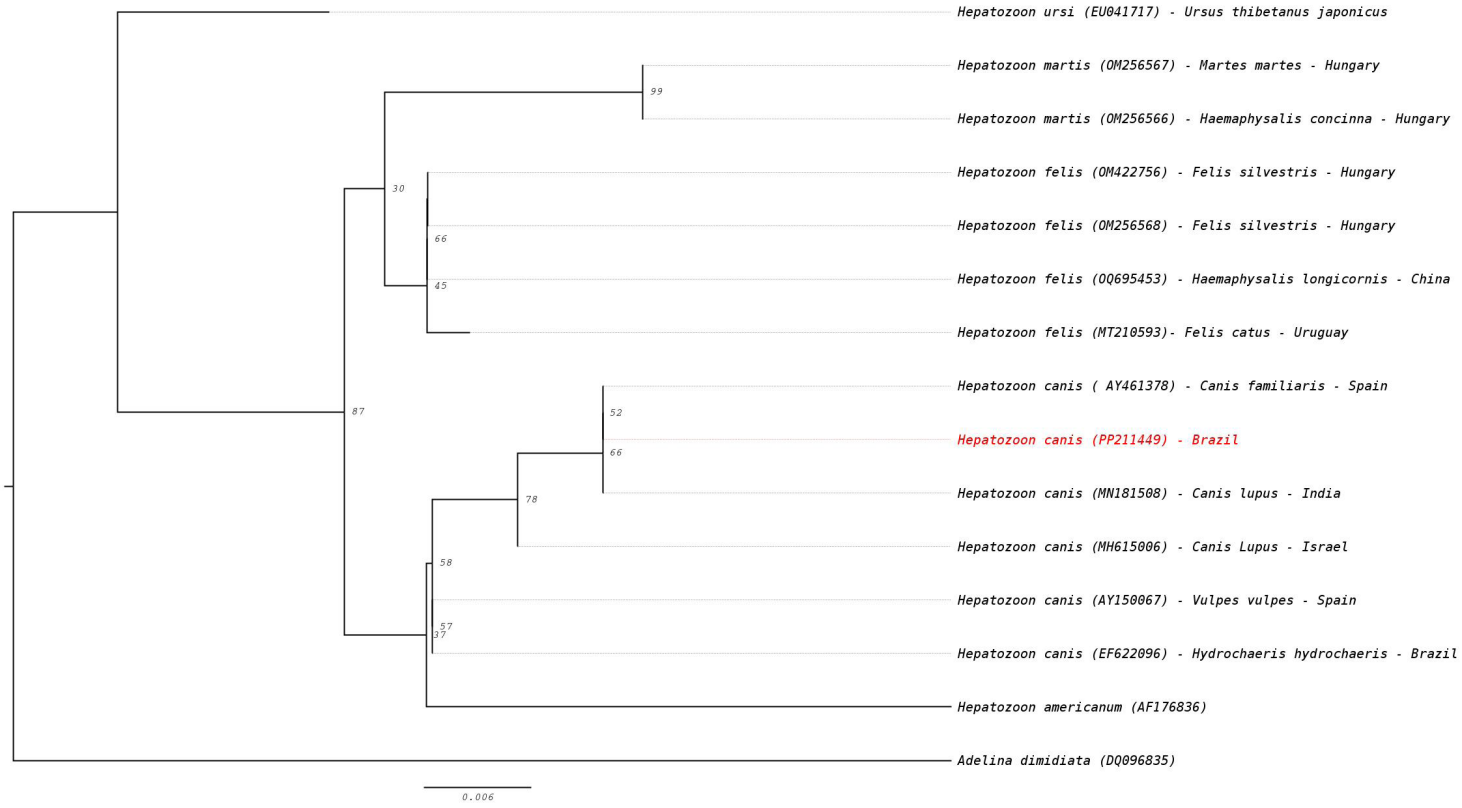
Phylogenetic tree based on the partial 18S rRNA gene sequences of *Hepatozoon canis* detected in hospital-treated dogs from a semi-arid region of Brazil, using the optimal model of substitution determined by ModelFinder implemented in IQ-TREE version 2.2.0.3. Accession numbers of the sequences obtained in this study and those from the GenBank database are indicated in parentheses.

Maximum likelihood phylogenetic analysis of the 18S rRNA gene showed that the *H. canis* sequence clustered with other *H. canis* lineages (accession numbers: AY461378 and MN181508), diverging from other species within the genus *Hepatozoon* (Figure 1).

**Figure 2:**
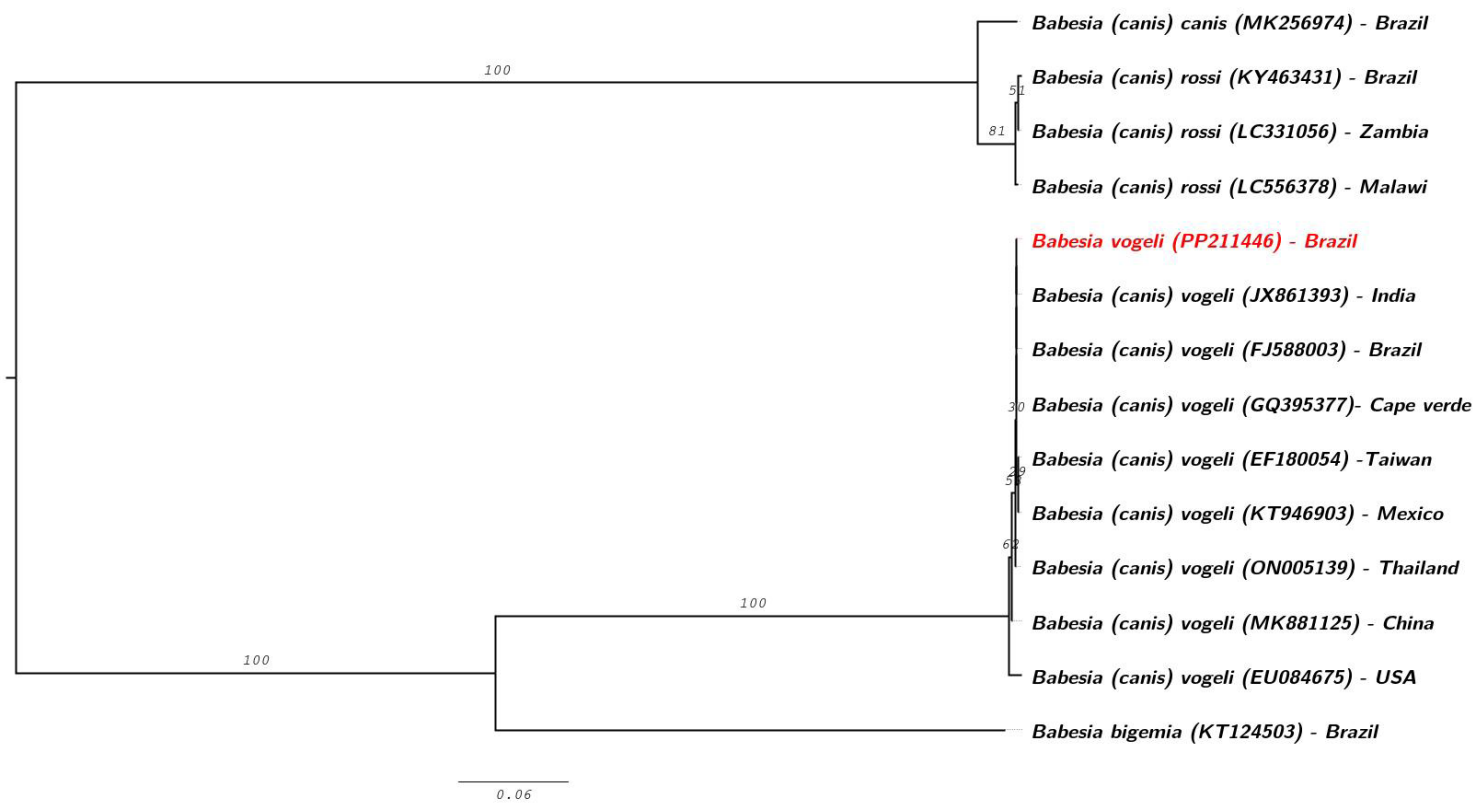
Phylogenetic tree based on the partial 18S rRNA gene sequences of *Babesia vogeli* detected in hospital-treated dogs from a semi-arid region of Brazil using the optimal model of substitution determined by ModelFinder implemented in IQ-TREE version 2.2.0.3. Accession numbers of the sequences obtained in this study and those from the GenBank database are indicated in parentheses.

*Babesia vogeli* sequence was grouped with other strains of the same species described in various geographical regions (India, Cape Verde, Taiwan, Mexico, Thailand, China, and USA) as well as with a sequence reported in Recife, Brazil (GenBank accession: FJ588003) (Figure 2).

**Figure 3:**
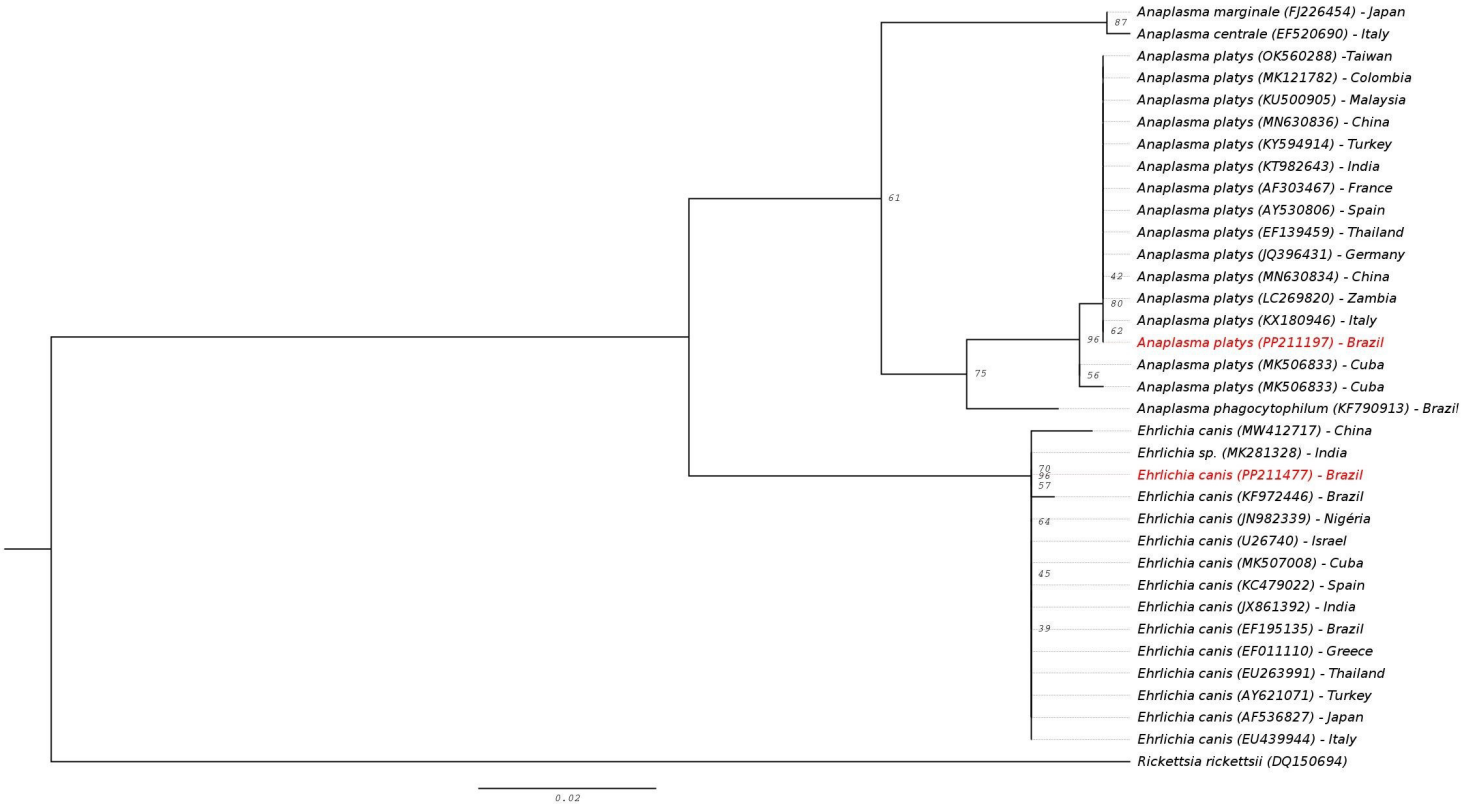
Phylogenetic tree based on partial 16S rRNA gene sequences of *Ehrlichia canis* and *Anaplasma platys* detected in hospital-treated dogs from a semi-arid region of Brazil, using the optimal substitution model determined by ModelFinder implemented in IQ-TREE version 2.2.0.3. Accession numbers of the sequences obtained in this study and those from the GenBank database are indicated in parentheses.

The 16S rRNA gene sequences from *E. canis* and *A. platys* demonstrated genetic similarity those of various strains reported worldwide (Figure 3).

## Discussion

This is the first and most extensive study to employ PCR as a diagnostic method to determine the frequency of single and multiple infections by *A. platys*, *B. vogeli*, *E. canis*, and *H. canis* and to phylogenetically characterize these agents in the state of Rio Grande do Norte in Brazil. Our results revealed a high frequency of tick-borne hemoparasites in dogs from a hospital population in the semi-arid region of Brazil, where approximately three out of every four dogs were infected with at least one of the pathogens studied. While 44.7% of these dogs were monoinfected, the frequencies of coinfections with two agents (21.5%) and with three or more agents (6.1%) were also notable.

The high frequency of these agents in the region may be attributable to several factors, including the characteristics of the study population, diagnostic method used, and presence and distribution of the vector. Specifically, the study was conducted in a population of dogs with suspected clinical signs of infections caused by tick-borne pathogens, which likely contributed to the high frequency observed. Additionally, the high sensitivity and specificity of the diagnostic method used play a crucial role in accurately detecting these infections.

The exposure of dogs to hemoparasitoses is known to be associated with increased activity of ixodid vectors in regions with favorable climatic and environmental conditions (Yabsley & Shock, 2013). The northeastern region of Brazil has climatic conditions conducive to the development of the vector tick *R. sanguineus* s.l. (Tanikawa et al., 2013), increasing the likelihood of animals being exposed to multiple infectious agents transmitted by this vector (Rotondano et al., 2017; Lopes et al., 2018). This underscores the need for further studies to determine the multiple pathogens transmitted by *R. sanguineus* s.l. within this region.

The use of PCR as a diagnostic method in this study enabled the identification of not only the exposure to the pathogens but also the cases of active infection by these agents, revealing the actual situation of these hemoparasitosites in the study population. Epidemiologically, the use of this molecular tool is important, as it enables the identification and distinction of healthy animals from those that serve as sources of infection for other vectors and animals, thereby facilitating targeted treatment of the involved pathogen.

The favorable climatic conditions for the presence and distribution of the vectors in the studied region may have also influenced the findings, considering that disease transmission by these vectors is typically directly linked to the vector (Dantas-Torres et al., 2020). Moreover, these conditions contribute to the exposure of animals to multiple types of hemoparasites because the abundance of ixodid ticks increases the risk of infection (Piantedosi et al., 2017; Di Cataldo et al., 2021). This aligns with the data obtained in our study, in which 84.62% of the animals coinfected with two agents were simultaneously parasitized by different bacterial and protozoan species.

Canine ehrlichiosis is considered one of the main infectious diseases in the clinical routine of small animals, likely due to the widespread presence of *R. sanguineus* s.l. throughout Brazil (Dantas-Torres et al., 2012). In this study, *E. canis* DNA was detected most frequently. *A. platys*, another bacterium of the order Rickettsiales, was the third most prevalent agent in the study population, highlighting the need for further research to better understand the epidemiology of these diseases in Rio Grande do Norte, Brazil. Notably, 90.9% (10/11) of the multiply infected animals tested positive for both *E. canis* and *A. platys* (Table 2). 

*Babesia vogeli* infection had the lowest detection rate among the studied pathogens in single infections (2.5%), coinfections (15.4%), and multiple infections (54.5%) (Table 2). The low occurrence of this agent in the northeastern region has been previously noted by other studies which employed PCR as a diagnostic method. For instance, Braga et al. (2019) reported a positivity rate of 4.8% (15/315) in dogs from a hospital population in the state of Piauí, Costa et al. (2015) a positivity of 0.9% (3/322) in canine populations from rural and urban areas in the interior of Maranhão, and Rotondano et al. (2015) a positivity of 10% (10/100) in dogs from a hospital population in Paraíba. Despite its low occurrence, periodic monitoring of this protozoan pathogen through epidemiological investigations is necessary, given that its prevalence can vary depending on the distribution of its vector, *R. sanguineus* s.l., and the increased mobility of hosts (Beugnet & Moreau, 2015).

Although the subclinical form of canine babesiosis is predominant in Brazil (Vidotto & Trapp, 2004), possibly due to the prevalence of *B. vogeli* species in the national territory, an immunocompromised status as well as cases of coinfection with other pathogens transmitted by *R. sanguineus* s.l. can exacerbate disease severity and adversely affect patient prognosis (Schnittger et al., 2012).

Data on the occurrence of canine hepatozoonosis in the semi-arid northeastern region of Brazil are scarce. The detection of *H. canis* in 35.4% of the dogs included in this study underscores the importance of this research, particularly because the pathogen was present in 74.4% of coinfection cases and 72.7% of multiple infection cases. These findings are significant due to the diverse clinical manifestations of the disease, which range from asymptomatic cases to various nonspecific clinical signs, including fever, lethargy, anemia, and muscle wasting, particularly in animals infected with multiple infectious agents. Furthermore, the chronic and debilitating nature of the disease may predispose dogs to concurrent infections with other pathogens (Baneth, 2011). This high prevalence of *Hepatozoon* spp. infections is particularly noteworthy given that it has not been commonly reported in this region before, suggesting emerging epidemiological changes (Margalit Levi et al., 2018).

Using PCR as a diagnostic method, Fonsêca et al. (2022) reported the occurrence of *H. canis* infection in 11.8% (18/153) of domiciled dogs in Jericoacoara (Ceará), similar to the results of Lopes et al. (2019), who detected *H. canis* DNA in 10% (2/20) of dogs from rural areas of Natal (Rio Grande do Norte). Canine hepatozoonosis is more prevalent in rural regions, likely because the causative pathogen has a natural sylvatic cycle and a higher chance of infestation of the affected mammals by vectors (Demoner et al., 2013). The increasing frequency of this pathogen in urban areas highlights the need for further investigations to clarify its epidemiological dynamics in these locations.

Clinical signs such as dehydration, inappetence, anorexia, weight loss, epistaxis, lethargy, fever, diarrhea, vomiting, pale mucous membranes, and lymphadenomegaly are frequently observed in dogs infected with *A. platys*, *E. canis*, *B. vogeli*, and *H. canis* (Bouzouraa et al., 2016; Zhang et al., 2023; Khalifa & Attia, 2024). These infections are also associated with hematological abnormalities, including anemia, thrombocytopenia, and, occasionally, leukopenia, with more severe manifestations often occurring in cases of multiple infections (Cevidanes et al., 2023). However, the clinical implications of coinfections involving *E. canis*, *A. platys*, *H. canis*, and *B. vogeli* remain poorly understood.

The various combinations of coinfections reported in this study underscore the need for further research into the epidemiological and clinicopathological aspects of naturally infected dogs. Interactions among these pathogens may exacerbate clinical signs, complicate diagnosis, and hinder the effectiveness of therapeutic approaches (Lara et al., 2020).

When comparing the results of direct pathogen detection in blood smears with those of molecular diagnostics (Table 3), a lower positivity in the former method was expected. Although simple and easy to perform, the direct identification of the agent on a slide offers low sensitivity and specificity. Particularly, it cannot distinguish between different species and may be influenced by the experience of the professional and the disease stage of the animal (Kaewkong et al., 2014).

Overall, when PCR is considered the reference method (gold standard), direct blood smear examination demonstrated an overall sensitivity of 30.1% and a specificity of 97.8% for detecting the hemoparasites *E. canis*, *H. canis*, *A. platys*, and *B. vogeli*, highlighting the superior diagnostic sensitivity and specificity of PCR compared to blood smear analysis. These results emphasize the importance of employing molecular methods, such as PCR, particularly in endemic regions and in scenarios of coinfection, where diagnostic accuracy is critical for appropriate clinical and therapeutic management.

The identification of pathogens via blood smear examination but not through molecular diagnosis may result from nonspecific intracellular inclusions. These inclusions in blood cells are often related to cellular activation during inflammatory processes and can be mistaken for other hemoparasites (Ferreira et al., 2007). Additionally, we cannot rule out the possibility of infection by other organisms of the order Rickettsiales, piroplasm species, and *Hepatozoon* spp., which were not tested in this study. The level of parasitemia should also be considered in cases wherein PCR amplification is absent (Gomes et al., 2016), and other variations of molecular techniques, such as nested PCR and real-time PCR (qPCR), are recommended in such cases because of their higher diagnostic sensitivity (Rufino et al., 2013).

For genotypic characterization of microorganisms through genetic sequencing, the use of genes that maintain a high degree of conservation within the evaluated genus and have long nucleotide sequences is recommended. The 16S rRNA gene used in this study for the phylogenetic characterization of the bacteria *E. canis* and *A. platys* was highly conserved, with low mutation frequencies. However, it is not the ideal gene for observing genetic variation within each species but is highly applicable for distinguishing between species (Dumler et al., 2001). This was also observed in our study, as demonstrated by the high similarity between the sequences of different strains described worldwide and those of *E. canis* and *A. platys* in this study.

Through partial sequencing of the 18S rRNA gene, which is commonly used for evolutionary studies of *Babesia* spp. and *Hepatozoon* spp. (Pinyoowong et al., 2008), we found significant sequence similarity between *H. canis* and *B. vogeli* samples obtained from Mossoró, located in the Brazilian semi-arid region, with reference samples for these species obtained from other regions of Brazil and worldwide. Despite having been extensively studied for these agents, this gene has limitations in molecular and phylogenetic studies due to the small sizes of the sequences generated, which can hinder a more detailed phylogenetic characterization depending on the application (Gomes et al., 2016; Modrý et al., 2017).

The presence of double peaks in the chromatographic analysis of some *H. canis* sequences not used for phylogenetic analysis in this study suggests coinfection by different isolates. However, molecular cloning techniques are necessary to characterize these sequences accurately.

The results of the molecular analyses conducted in this study contribute significantly to the understanding of the etiology and geographic distribution of *A. platys*, *E. canis*, *B. vogeli*, and *H. canis* in northeastern Brazil. This is particularly noteworthy because of the absence of previous studies that provide molecular characterization data for these pathogens in the studied region.

Despite the robust sample size and the use of sensitive molecular methods, this study has limitations that should be acknowledged. Specifically, a convenience sampling strategy was employed, selecting dogs based on clinical and/or laboratory suspicion of tick-borne diseases. While this approach facilitated the detection of active cases, it may introduce selection bias and limit the generalizability of the findings to the broader canine population, particularly asymptomatic animals. Nevertheless, this strategy was necessary to capture clinically relevant infections and enable the genetic characterization of the pathogens involved. Although the present findings provide valuable insights into the frequency and molecular aspects of tick-borne infections in symptomatic dogs from the study region, further studies employing randomized or stratified sampling designs are recommended to complement and expand these results, allowing comparisons across diverse epidemiological contexts, including asymptomatic populations.

## Conclusions

This is the first comprehensive study in the state of Rio Grande do Norte, Brazil, to simultaneously investigate infections by *Anaplasma platys*, *Ehrlichia canis*, *Babesia vogeli*, and *Hepatozoon canis* using PCR as a diagnostic tool, combined with phylogenetic characterization of the detected agents. Although previous studies have reported some of these pathogens in the same region — including in the same municipality — none have encompassed all four agents with this level of molecular detail. We found a high prevalence of hemoparasites in the study population, with *E. canis* being the most common, followed by *H. canis*, *A. platys*, and *B. vogeli*. Coinfections were frequently observed, highlighting the need for comprehensive diagnostic approaches that consider multiple tick-borne pathogens in endemic areas. The occurrence of multiple infections can worsen clinical outcomes, complicate treatment, and negatively affect prognosis. Early and accurate diagnosis, along with preventive measures, is crucial for reducing infection rates in dogs and limiting the spread of these pathogens to other tick vectors.

## Data Availability

Data will be made available on request.
